# Fabrication and Characterization of a Trans‐Activator of Transcription Peptide–Functionalized Collagen–Mineral Trioxide Aggregate Nanofiber Scaffold: Physicochemical Properties and Fibroblast Response

**DOI:** 10.1002/cre2.70437

**Published:** 2026-08-19

**Authors:** Mehdi Sheikharabi, Javad Delkabadi, Ayyoob Khosravi, Ezatolah Kazeminejad

**Affiliations:** ^1^ Department of Medical Biotechnology, Faculty of Advanced Technologies Golestan University of Medical Sciences Gorgan Iran; ^2^ Department of Radiology, Faculty of Dentistry Mashhad University of Medical Sciences Mashhad Iran; ^3^ Stem Cell Research Center, Biomedical Research Institute Golestan University of Medical Sciences Gorgan Iran; ^4^ Dental Research Center, Jorjani Clinical Sciences Research Institute Golestan University of Medical Sciences Gorgan Iran

**Keywords:** collagen, electrospinning, endodontics, mineral trioxide aggregate, poly(ε‐caprolactone), TAT peptide, tissue engineering

## Abstract

**Objectives:**

Electrospun nanofibrous scaffolds that mimic extracellular matrix (ECM) architecture are promising for endodontic and craniofacial tissue engineering. This study aimed to fabricate a composite electrospun scaffold comprising poly(ε‐caprolactone) (PCL), collagen, mineral trioxide aggregate (MTA), and a TAT cell‐penetrating peptide, and to evaluate its physicochemical properties and short‐term cytocompatibility with fibroblasts.

**Material and Methods:**

Electrospun mats were prepared from PCL and PCL–collagen blends with or without incorporation of MTA and TAT peptide. Fiber morphology and diameter were assessed by scanning electron microscopy (SEM). Chemical structure was analyzed using Fourier‐transform infrared (FTIR) spectroscopy. Surface charge was determined by zeta potential measurement before and after TAT functionalization. Tensile properties of the PCL–collagen–MTA–TAT scaffold were evaluated using uniaxial testing. Cytotoxicity toward human fibroblasts was assessed using the MTT assay after 24 and 48 h of culture on PCL, PCL–collagen, PCL–collagen–MTA, and PCL–collagen–MTA–TAT scaffolds. Additional physicochemical characterization included water contact angle and biodegradability.

**Results:**

SEM revealed continuous, bead‐free fibers forming a porous, interconnected network with submicron diameters in all groups. FTIR spectra confirmed the presence of the expected components in the composite scaffold. Zeta potential shifted from negative values in PCL–collagen–MTA scaffolds to positive values after TAT incorporation, indicating surface modification. The PCL–collagen–MTA–TAT scaffold exhibited tensile behavior compatible with handling. MTT results showed fibroblast viability exceeding 90% for all scaffold compositions at both time points relative to tissue culture plastic controls, with no evidence of cytotoxicity. Water contact angle and biodegradability suggested that incorporation of collagen, MTA, and TAT improved scaffold surface characteristics.

**Conclusions:**

The electrospun PCL–collagen–MTA–TAT scaffold demonstrated ECM‐like morphology, modified surface charge, acceptable handling properties, and short‐term fibroblast compatibility. This composite nanofibrous system may serve as a preliminary platform for further investigation in bone and dental tissue engineering; however, additional functional and long‐term biological studies are required to confirm its regenerative potential.

## 1 Introduction

Bone regeneration and repair are dynamic physiological processes that depend on a complex interplay between cells, extracellular matrix (ECM), and signaling molecules to restore structural integrity and function after injury or disease (Asghari et al. 2017; Vallet‐Regí and Ruiz‐Hernández 2011). In the craniofacial region, large bony defects resulting from trauma, infection, tumors, congenital anomalies, or extensive periapical lesions may exceed the host's intrinsic regenerative capacity, necessitating the use of bone substitutes or tissue‐engineered constructs to guide and enhance healing (Qu et al. 2019; Qi et al. 2021; Pace et al. 2025). Conventional grafting techniques based on autografts, allografts, and xenografts have improved clinical outcomes. Still, they remain associated with significant limitations, including donor‐site morbidity, limited availability of graft material, risk of disease transmission, immunological reactions, and unpredictable resorption (Qu et al. 2019; Iqbal et al. 2019). These drawbacks have highlighted the need for synthetic and hybrid biomaterials that can act as scaffolds to support cell adhesion, proliferation, and differentiation, ultimately promoting predictable regeneration of bone and related mineralized tissues (Yuan et al. 2019; Rahimkhoei et al. 2023).

An ideal scaffold for bone tissue engineering should be biocompatible, bioactive, and biodegradable, with a degradation rate that matches the rate of new tissue formation (Qu et al. 2019; Iqbal et al. 2019). It must provide sufficient mechanical strength to withstand physiological loads while maintaining a highly porous and interconnected structure that facilitates cell migration, neovascularization, and the diffusion of nutrients and metabolic waste (Pace et al. 2025; Yuan et al. 2019). Importantly, the scaffold should recapitulate the structural and biochemical features of the native ECM, which consists of fibrous proteins, proteoglycans, and glycosaminoglycans arranged in a hierarchical architecture (Qi et al. 2021; Rahimkhoei et al. 2023). This ECM not only provides mechanical support but also delivers essential biochemical cues that modulate cell morphology, cytoskeletal organization, and gene expression, thereby directing lineage commitment and functional maturation. Reproducing this microenvironment with biomimetic scaffolds is therefore a central objective of contemporary tissue engineering strategies (Asghari et al. 2017; Qi et al. 2021; Pace et al. 2025).

Among biodegradable synthetic polymers, poly(ε‐caprolactone) (PCL) has emerged as a widely used material for scaffold fabrication due to its favorable mechanical properties, ease of processing, and relatively low cost (Backes et al. 2022; Liang et al. 2024). PCL is an aliphatic polyester that degrades slowly by hydrolysis of its ester bonds under physiological conditions, providing long‐term mechanical stability and allowing sufficient time for new bone formation (Backes et al. 2022; Zhu 2022). Its biocompatibility has been demonstrated in various in vitro and in vivo models, making it particularly attractive for musculoskeletal and craniofacial applications (Backes et al. 2022; Liang et al. 2024). However, PCL is intrinsically hydrophobic and lacks specific biological recognition sites, which can limit protein adsorption and cell adhesion when used alone (Liang et al. 2024; Zhu et al. 2022). To overcome these shortcomings, PCL is frequently combined with natural polymers or bioactive phases to enhance its surface properties and biological performance, thereby creating composite scaffolds that more closely resemble the natural ECM (Iqbal et al. 2019; Rahimkhoei et al. 2023; Backes 2022; Liang et al. 2024; Zhu et al. 2022).

Collagen, the most abundant protein in the human body, is the predominant organic component of bone, dentin, and periodontal tissues (Li et al. 2021). Type I collagen forms fibrillar networks that confer tensile strength and provide an organized three‐dimensional microenvironment for osteoblasts, fibroblasts, and other cells involved in tissue remodeling and regeneration (Li et al. 2021; Zhang et al. 2023). Beyond its mechanical role, collagen contains specific peptide sequences that act as signaling motifs, regulating cell attachment, migration, proliferation, and differentiation (Zhang et al. 2023; Wang et al. 2023; Valverde et al. 2016). Collagen‐based biomaterials have thus been extensively investigated as scaffolds, membranes, and matrices for bone and dental tissue engineering, demonstrating good short‐term biocompatibility and low immunogenicity (Wang et al. 2023; Valverde et al. 2016; Rico‐Llanos et al. 2021). Incorporating collagen into PCL scaffolds can improve hydrophilicity, increase protein adsorption, and supply bioactive cues that support osteogenic and fibroblastic responses, bringing the composite closer to the native ECM of mineralized tissues (Li et al. 2021; Valverde et al. 2016; Rico‐Llanos et al. 2021).

Mineral trioxide aggregate (MTA) is a hydrophilic, bioactive endodontic cement that has been widely used in root‐end filling, perforation repair, pulp capping, and regenerative endodontic procedures due to its excellent sealing ability and biocompatibility (Tu et al. 2019; Serin Kalay 2019). Chemically, MTA is composed primarily of tricalcium silicate, dicalcium silicate, tricalcium aluminate, and bismuth oxide, and it sets in the presence of moisture to form a calcium silicate hydrate matrix and calcium hydroxide (Tu et al. 2019; Serin Kalay 2019; Lopes et al. 2019). MTA has been shown to stimulate biomineralization and promote the formation of hard tissue barriers, partly through sustained calcium ion release and the creation of an alkaline microenvironment that favors apatite deposition (Tu et al. 2019; Serin Kalay 2019; Lopes et al. 2019). Experimental studies indicate that MTA can upregulate the expression of bone morphogenetic protein‐2 (BMP‐2) and dentin matrix acidic phosphoprotein‐1 (DMP1), thereby enhancing the differentiation of progenitor cells into osteoblastic and odontoblastic lineages (Lopes et al. 2019; Yamada et al. 2021). These osteoconductive and osteoinductive properties make MTA an attractive additive in polymeric scaffolds designed for bone and periapical tissue regeneration (Tu et al. 2019; Serin Kalay 2019; Lopes et al. 2019; Yamada et al. 2021).

In addition to osteoconductive and osteoinductive components, strategies that improve cell–scaffold interactions at the surface level can substantially enhance the performance of biomaterials. In molecular biology, the trans‐activator of transcription (TAT) protein, encoded by human immunodeficiency virus (HIV), is a regulatory protein that facilitates viral transcription and has a remarkable ability to translocate across cell membranes (Xie et al. 2020; Debaisieux et al. 2012; Guidotti et al. 2017). Short peptide fragments derived from TAT, commonly referred to as TAT peptides, belong to the family of cell‐penetrating peptides (CPPs) and are characterized by a high content of basic amino acids such as arginine and lysine, resulting in a strong positive charge at physiological pH (Debaisieux et al. 2012; Guidotti et al. 2017; Jiang et al. 2018; Datta 2025). This cationic nature enables electrostatic interactions with negatively charged cell membranes and promotes rapid cellular uptake of TAT‐conjugated cargos (Debaisieux et al. 2012; Guidotti et al. 2017; Jiang et al. 2018; Datta 2025). When immobilized on biomaterial surfaces, TAT peptides can increase cell adhesion, spreading, and proliferation by enhancing the affinity between cells and the scaffold and by modulating the adsorption of serum proteins (Guidotti et al. 2017; Jiang et al. 2018; Datta 2025). These properties suggest that TAT functionalization of nanofibrous scaffolds may be a powerful approach to improving the bioactivity of constructs intended for bone and dental tissue engineering.

Electrospinning is a versatile and relatively simple technique for producing continuous fibers with diameters from the micrometer range down to a few tens of nanometers using a high‐voltage electric field applied to a polymer solution or melt (Epicoco et al. 2023). The resulting nonwoven mats typically possess a high surface‐area‐to‐volume ratio, tunable porosity, and fiber diameters comparable to those of natural collagen fibrils, closely mimicking the architecture of native ECM (Epicoco et al. 2023). Such electrospun nanofibrous scaffolds have demonstrated significant potential for applications in skin, cartilage, nerve, and bone regeneration, as well as in controlled drug delivery (Epicoco et al. 2023). For endodontic and maxillofacial applications, electrospinning enables fabrication of PCL–collagen composite scaffolds loaded with bioactive components such as MTA and functionalized with the TAT peptide, resulting in a composite scaffold that incorporates multiple components and combines mechanical stability, biomimetic structure, osteogenic signaling, and enhanced cell adhesion.

In this context, designing a nanofiber scaffold that integrates the mechanical advantages of PCL, the biological instructiveness of collagen, the osteogenic potential of MTA, and the cell‐adhesive properties of the TAT peptide may offer a strategy for craniofacial and endodontic tissue engineering. Such a scaffold has the potential to support bone regeneration in periapical defects and other challenging clinical situations in which conventional materials are inadequate. However, before translation into in vivo or clinical settings, comprehensive characterization of the scaffold's physicochemical properties, surface charge, mechanical behavior, and cytocompatibility is required. Therefore, the present study aimed to fabricate and characterize an electrospun nanofiber scaffold composed of PCL, collagen, MTA, and the TAT peptide, and to evaluate its cytocompatibility and initial cellular response. The working hypothesis was that the PCL–collagen–MTA–TAT nanofiber scaffold would exhibit suitable morphology, surface charge, and mechanical properties for tissue engineering applications and would support cytocompatibility and initial cell response.

## 2 Materials and Methods

### 2.1 Materials

**Figure 1:**
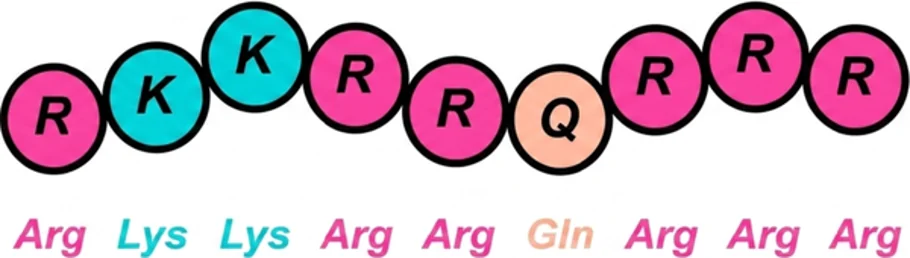
TAT peptide sequence.

Polycaprolactone (PCL, Sigma‐Aldrich, Mn ≈ 80,000), type I collagen (Collagino), MTA (Cerkamed, Poland), and TAT peptide (Figure 1) were used as received. Analytical‐grade acetic acid (Merck), chloroform (Merck), and dimethylformamide (DMF, Merck) were used as solvents. The human dermal fibroblast (HDF) cell line was obtained from the National Cell Bank of Iran at the Pasteur Institute (NCBI code: C645) and cultured in Dulbecco's Modified Eagle's Medium (DMEM/F12, BIO‐IDEA; Cat. Number BI‐1012). Fetal bovine serum (FBS, BIO‐IDEA), penicillin–streptomycin, trypsin, phosphate‐buffered saline (PBS, BIO‐IDEA), MTT powder, and dimethyl sulfoxide (DMSO) were used for cytotoxicity testing.

### 2.2 Preparation of Electrospun Scaffolds

Five types of electrospun mats were fabricated for subsequent characterization and biological testing: (1) PCL; (2) PCL–collagen blends with PCL: collagen weight ratios of 90:10, 80:20, and 70:30; (3) PCL–collagen–MTA (PCL: collagen 90:10 containing MTA); and (4) PCL–collagen–MTA–TAT peptide (final target scaffold).

For all formulations, 1.36 g of PCL was dissolved in a solvent mixture consisting of 1.5 mL DMF, 1.5 mL acetic acid, and 7 mL chloroform under vigorous magnetic stirring at room temperature for 1 h, followed by slower stirring for 30 min to obtain a homogeneous solution. For PCL–collagen blends, collagen was added to the PCL solution at 0.14, 0.34, and 0.58 g to obtain PCL: collagen ratios of 90:10, 80:20, and 70:30 (w/w), respectively.

For the PCL–collagen–MTA scaffold, 0.14 g collagen and 0.05 g MTA were added to the PCL solution; for the PCL–collagen–MTA–TAT scaffold, 0.14 g collagen, 0.05 g MTA, and 0.05 g TAT peptide were added to the PCL solution. All mixtures were stirred vigorously for 1 h and then gently for 30 min at room temperature to ensure complete dispersion.

TAT peptide was incorporated via physical blending without covalent bonding. While this approach enables effective surface charge modulation, it may affect long‐term peptide stability, warranting further investigation.

Each solution was loaded into a 10‐mL syringe fitted with a metallic 18‐gauge needle and electrospun using a custom electrospinning setup. Electrospinning was performed at a flow rate of 0.1 mL/min, an applied voltage of 17 kV, and a tip‐to‐collector distance of 15 cm. Fibers were collected on aluminum foil fixed to a grounded rotating collector rotating at 500 rpm.

### 2.3 Fourier‐Transform Infrared Spectroscopy (FTIR)

The chemical structures and functional groups of PCL, collagen, the TAT peptide, and the final PCL–collagen–MTA–TAT scaffold were investigated using FTIR spectroscopy. Thin films of the electrospun mats were ground with KBr, compressed into pellets, and analyzed over the wavenumber range 400–4000 cm^−1^.

### 2.4 Scanning Electron Microscopy (SEM)

Morphology and fiber diameter of the electrospun scaffolds were examined using a SEM. Square specimens (1 × 1 cm^2^) were cut from each mat, mounted on stubs, and sputter‐coated with a thin layer of gold before imaging. Fiber diameters were measured from SEM micrographs using image‐analysis software, and at least 50 fibers per image were analyzed to obtain the mean diameter.

### 2.5 Zeta Potential Measurement

To determine the surface charge, the zeta potential was measured for PCL–collagen–MTA scaffolds before and after incorporating the TAT peptide. Suspensions of the electrospun fibers were prepared in distilled water and adjusted to pH 7. The zeta potential was determined using a zeta potential analyzer.

### 2.6 Mechanical Testing

Tensile properties of the PCL–collagen–MTA–TAT scaffolds were evaluated using a universal testing machine to assess mechanical behavior relevant to scaffold handling. Rectangular strips (1 × 3 cm^2^) were cut from the mats and tested in uniaxial tension at a crosshead speed of 5 mm/min. Stress–strain curves were recorded, and the elastic modulus (from the linear region), ultimate tensile strength, and strain at break were calculated.

### 2.7 Cell Culture

HDFs were cultured in DMEM supplemented with 20% FBS and 1% penicillin–streptomycin. Cells were maintained in T‐25 flasks at 37°C in a humidified atmosphere containing 5% CO_2_. For subculture, cells were rinsed with PBS, detached using trypsin, neutralized with complete medium, centrifuged, resuspended, and seeded into new flasks. Cell counting was performed using a hemocytometer and trypan blue exclusion to assess viability.

### 2.8 Scaffold Sterilization and Seeding

Electrospun discs were punched to fit the wells of 96‐well plates. Before cell seeding, scaffolds were washed with 70% ethanol for 3 h to disinfect them and then exposed to ultraviolet light for 20 min. Scaffolds were placed at the bottom of each well, and 10,000 fibroblast cells were seeded onto each disc in complete medium. Plates were incubated at 37°C and 5% CO_2_ for 24 and 48 h. Experimental groups included PCL, PCL–collagen (90:10, 80:20, and 70:30), PCL–collagen–MTA, and PCL–collagen–MTA–TAT; wells containing cells without scaffolds served as controls.

### 2.9 MTT Assay for Cell Viability

Cell viability and cytotoxicity of the scaffolds toward fibroblasts were evaluated using the MTT assay. After 24 and 48 h of culture, the culture medium in each well was removed, and MTT solution was added. Plates were incubated for 4 h at 37°C to allow formation of formazan crystals in metabolically active cells. Subsequently, DMSO was added to each well to dissolve the crystals, and the optical density was measured at 570 nm using a microplate reader. Cell viability was expressed as a percentage relative to the control group (cells cultured on tissue‐culture plastic without a scaffold). Four replicate wells were used for each sample and time point.

### 2.10 Biodegradability Test

The biodegradability test is a key evaluation for assessing the long‐term performance of nanofibrous scaffolds in biological environments. This test examines the rate and extent of nanofiber degradation under simulated physiological conditions. The results indicate whether the scaffold can safely degrade in the body after fulfilling its biological function (e.g., supporting new tissue growth).

This test was conducted in accordance with ASTM F1635. Samples measuring 1 × 1 cm were prepared, and their initial dry weight (Ww) was measured using a digital balance with an accuracy of ±0.1 mg. The samples were immersed in 3 mL of PBS solution with a pH of 7.3 and incubated at 37°C for 14 days.

At predetermined time intervals (days 1, 7, and 14), the samples were removed and dried in an oven at 40°C for 4 h to eliminate moisture. The remaining weight (Wr) was then measured and recorded. The biodegradability percentage was calculated using the appropriate equation and reported.

The samples evaluated in this test included: (1) nanofibers composed of PCL and (2) nanofibers composed of PCL–collagen–MTA–TAT with a ratio of 90:10.

### 2.11 Water Contact Angle

This technique is a widely used and important method for evaluating the surface properties of nanofibers, particularly for determining their hydrophilicity or hydrophobicity. In this study, the water contact angle test was performed using a surface contact angle measurement device (CAG‐10, Zhikan, Iran) at the Gorgan University of Agricultural Sciences and Natural Resources.

According to the device protocol and the ISO 19403‐2:2017 standard, a droplet of distilled water (3–5 μL) was gently placed on the flat surface of the sample. The contact angle was then measured and recorded using a digital imaging system and image analysis software. All measurements were conducted at room temperature under controlled environmental conditions.

This analysis was performed both before and after plasma surface modification of nanofibers composed of PCL–collagen–MTA–TAT at a 90:10 ratio.

To improve hydrophilicity and facilitate cellular infiltration, the nanofiber samples were placed in a Diener plasma system (manufactured in Germany; supplied by Bonyakhteh Technology Company, Tehran). This process was carried out using pure oxygen gas at a pressure of 0.80 mbar and a power of 60 W for 5 min.

### 2.12 Statistical Analysis

All quantitative data are presented as mean ± standard deviation. The effects of scaffold composition and incubation time on cell viability were analyzed using factorial analysis of variance (ANOVA) in a completely randomized design. A significance level of *p* < 0.05 was adopted for all comparisons. Where applicable, statistical differences are reported in relation to the control and between scaffold groups. The present study was designed as a preliminary investigation; therefore, no formal power analysis was performed.

### 2.13 Ethical Considerations

The study protocol for the use of human fibroblast cell lines was reviewed and approved by the institutional Ethics Committee.

## 3 Results

### 3.1 Morphology and Fiber Diameter of Electrospun Scaffolds

**Figure 2:**
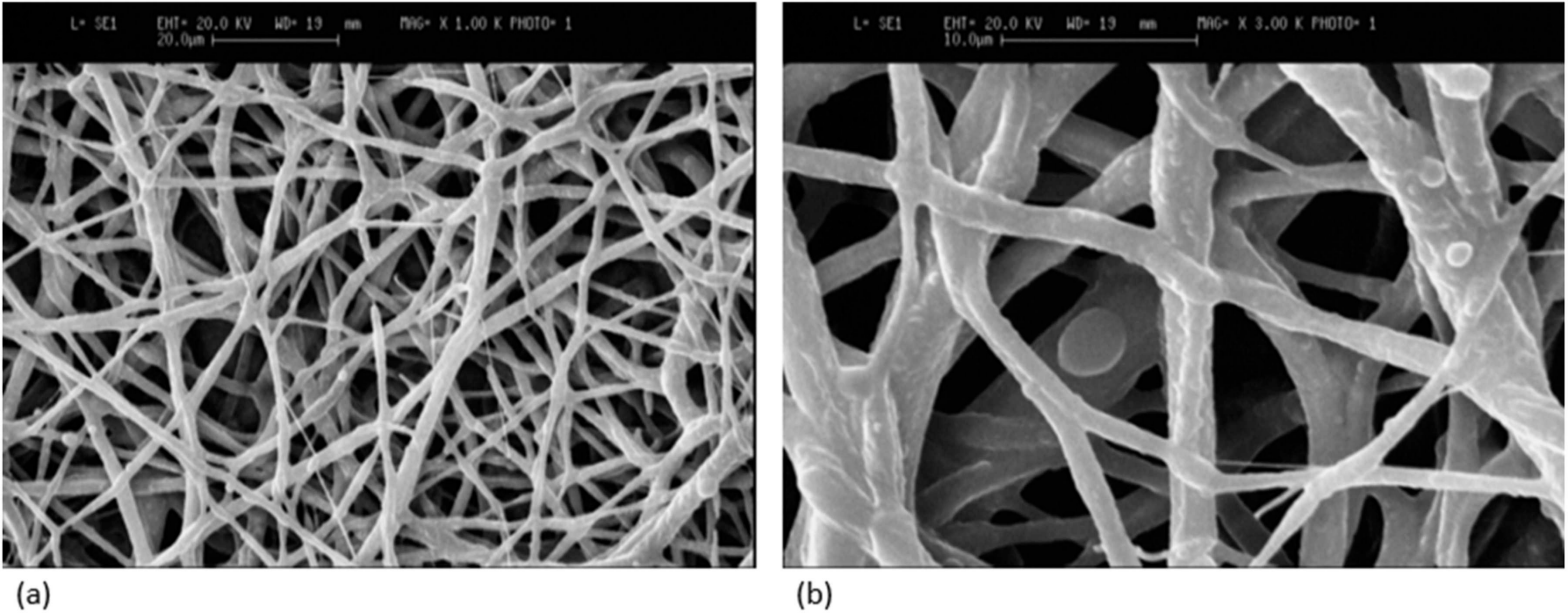
SEM morphology of electrospun nanofibrous scaffolds. (a) An interconnected, randomly oriented porous fibrous network at ×1000 magnification (scale bar: 20 μm); and (b) detailed fiber morphology, inter‐fiber junctions, and discrete surface‐associated particulate deposits at ×3000 magnification (scale bar: 10 μm).

Scanning electron microscopy (SEM) images revealed that all electrospun scaffolds exhibited continuous, bead‐free fibers that formed a highly porous, interconnected nanofibrous network (Figure 2). No morphological defects or fiber fusion were observed in any of the formulations, indicating stable electrospinning conditions for all compositions. Quantitative analysis of fiber diameters using ImageJ software showed that the mean fiber diameter of the scaffolds was approximately 130 nm, which is considered favorable for ECM mimicry and cell–material interactions. Incorporation of collagen and MTA did not result in significant fiber irregularities, while the addition of TAT peptide did not adversely affect fiber uniformity. These morphological findings are consistent with successful electrospinning of the scaffold formulations. Overall, the nanofibrous architecture closely resembled native ECM structures and provided a high surface‐area‐to‐volume ratio suitable for tissue engineering applications.

### 3.2 Chemical Characterization by FTIR

**Figure 3:**
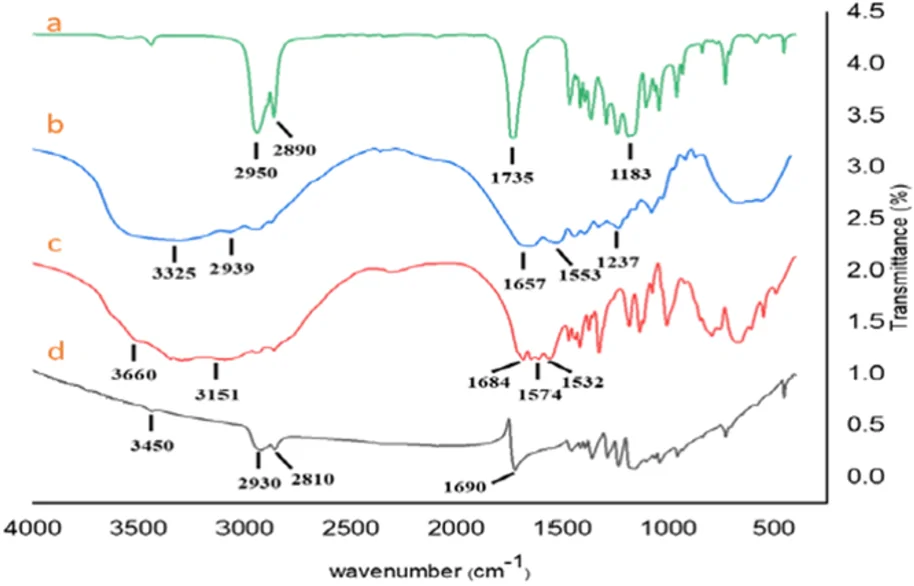
FTIR spectra of scaffold components and composite nanofibers.

FTIR spectra confirmed the successful incorporation of all intended components within the composite scaffold (Figure 3). The characteristic absorption bands of PCL were preserved in the composite nanofibers, indicating that the polymer backbone remained chemically stable during electrospinning. Distinct amide I and amide II bands corresponding to collagen were observed in the PCL–collagen–MTA–TAT scaffold, confirming the presence of the protein component. Additional spectral features consistent with the incorporation of MTA and TAT peptides were detected in the composite scaffold. The available FTIR data primarily provide qualitative confirmation of scaffold composition.

### 3.3 Surface Charge Analysis

**Figure 4:**
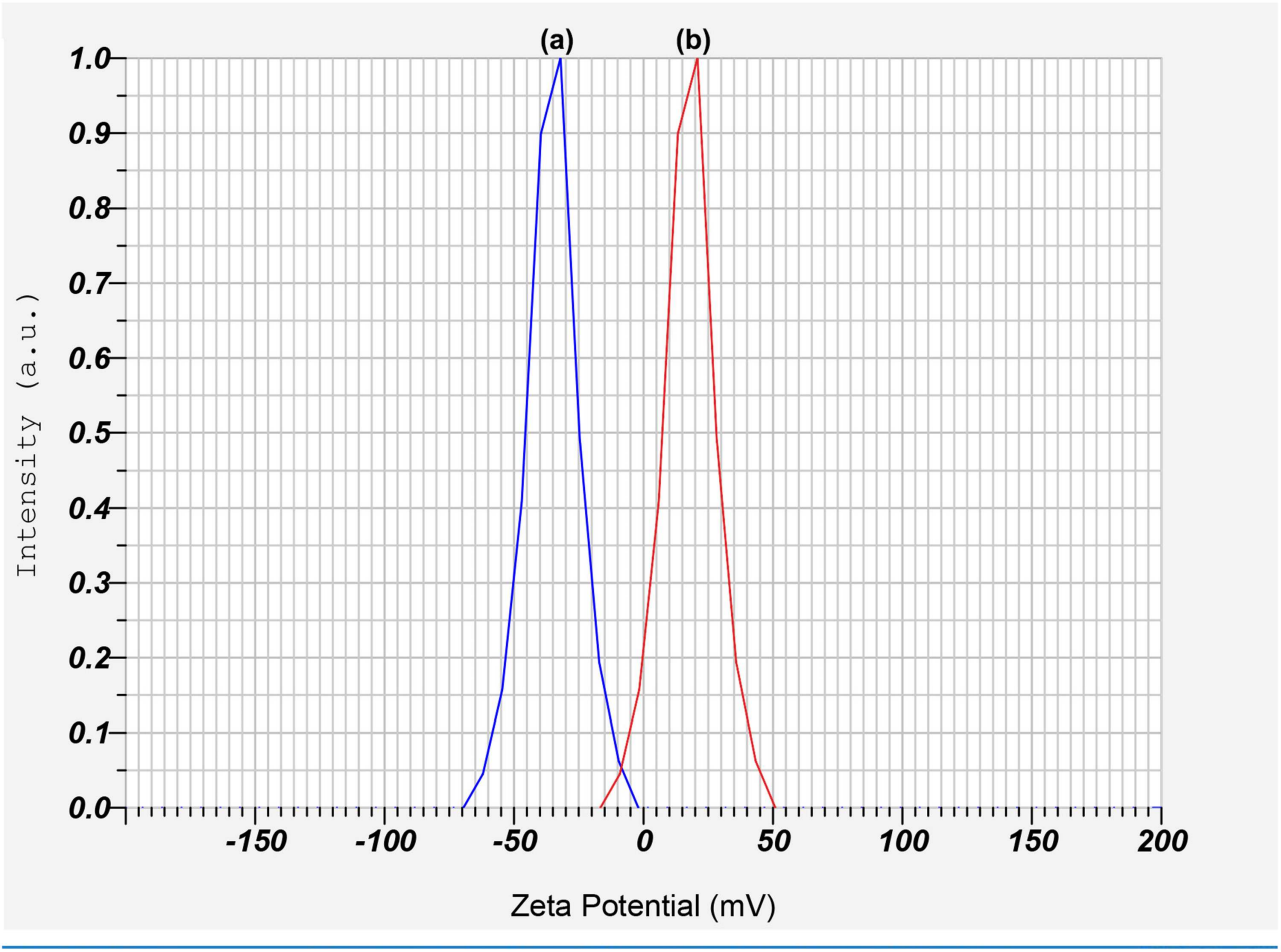
Zeta potential of composite scaffolds before (a) and after (b) TAT peptide modification.

Zeta potential measurements demonstrated a clear change in surface charge following TAT peptide incorporation (Figure 4, Table 1). The PCL–collagen–MTA scaffold exhibited a negative surface charge (approximately −30 mV) at physiological pH. After functionalization with TAT peptide, the surface charge shifted to a positive value (approximately +20 mV), indicating successful surface modification. This inversion of surface charge is attributed to the high density of basic amino acids present in the TAT peptide. It confirms effective exposure of cationic functional groups on the fiber surface. Such modulation of surface charge is expected to influence protein adsorption and subsequent cell–scaffold interactions.

**Table 1:** Zeta potential of composite scaffolds before and after TAT peptide functionalization.

Scaffold group	Zeta potential (mV)
PCL–collagen–MTA	−30
PCL–collagen–MTA–TAT peptide	+20

### 3.4 Mechanical Properties

**Figure 5:**
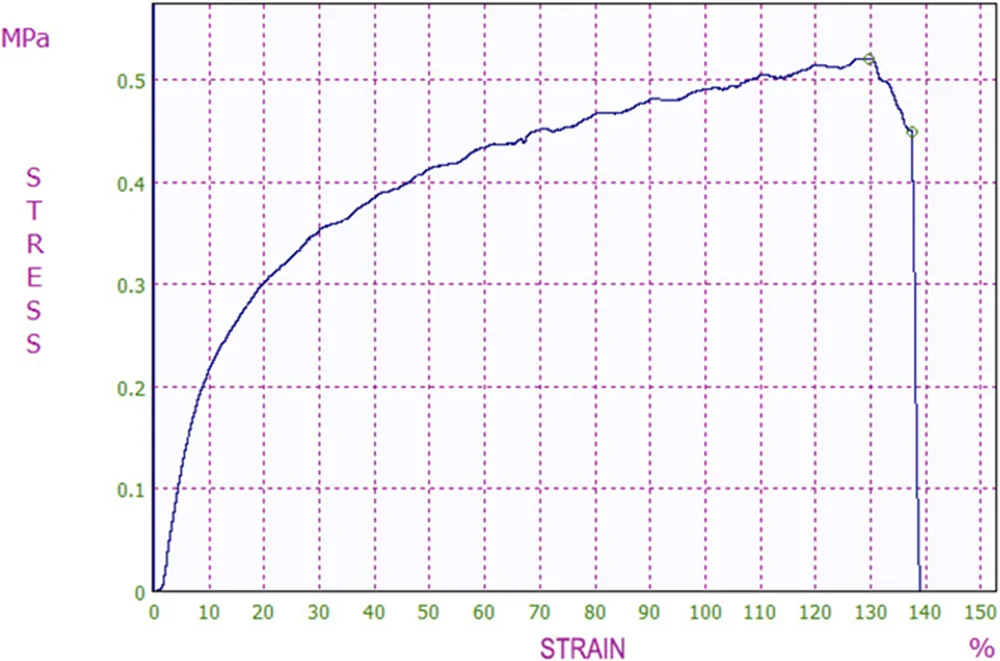
Tensile stress–strain behavior of the PCL–collagen–MTA–TAT peptide scaffold.

Uniaxial tensile testing revealed that the electrospun PCL–collagen–MTA–TAT scaffold exhibited mechanical behavior characteristic of ductile PCL‐based nanofibrous mats (Figure 5). The stress–strain curve displayed an initial linear elastic region followed by plastic deformation before failure. The elastic modulus, ultimate tensile strength, and strain at break of the composite scaffold fell within the range reported for electrospun PCL‐based scaffolds intended for bone and soft tissue regeneration (Table 2). The PCL–collagen–MTA–TAT scaffold exhibited tensile behavior that supported handling under the test conditions. These results provide preliminary mechanical characterization, but further optimization and comparison with tissue‐specific benchmarks are required before clinical relevance can be inferred.

**Table 2:** Tensile properties of the PCL–collagen–MTA–TAT peptide scaffold.

Sample	Elastic modulus (MPa)	Breaking strain (%)	Ultimate tensile strength (MPa)	Tensile stress (MPa)	Tensile strain (%)
PCL–COLL–MTA–TAT peptide	0.402	137	13.07	0.52	129

### 3.5 Fibroblast Viability and Cytocompatibility

**Figure 6:**
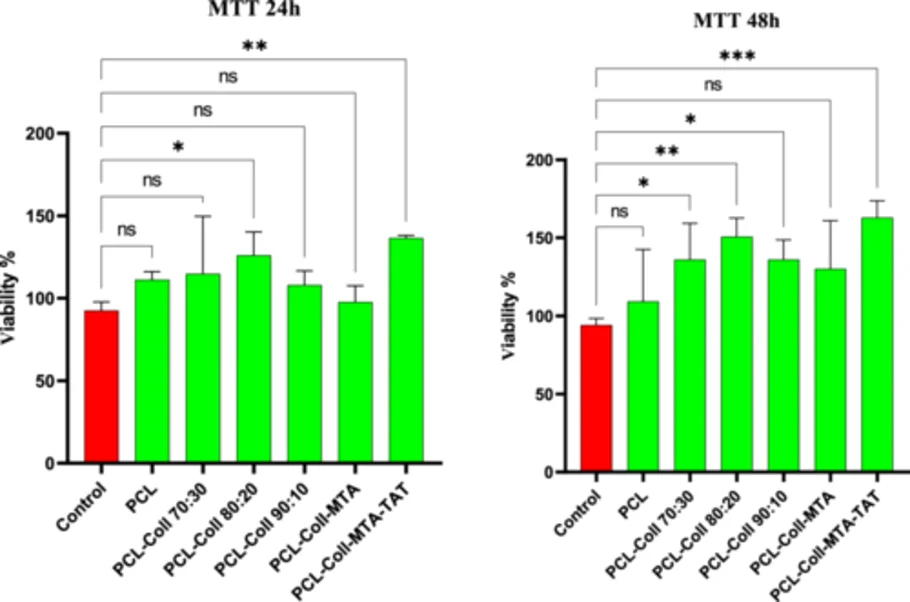
Cell viability of fibroblasts cultured on electrospun scaffolds. ns: non significant *p < 0/1, **p < 0/01, ***p < 0/001.

The cytocompatibility of the electrospun scaffolds was evaluated using the MTT assay after 24 and 48 h of culture (Figure 6). All scaffold groups—including PCL, PCL–collagen blends, PCL–collagen–MTA, and PCL–collagen–MTA–TAT—exhibited high levels of fibroblast viability relative to tissue culture plastic controls. At both time points, cell viability exceeded 90% for all scaffold compositions, indicating the absence of cytotoxic effects. Statistical analysis showed no significant reduction in viability for any scaffold group compared with the control at 24 h (*p* > 0.05). Although minor statistically significant differences among some groups were observed at 48 h, all values remained within a range compatible with good cytocompatibility. Notably, in this short‐term assay, the PCL–collagen–MTA–TAT scaffold did not exhibit any adverse effect on fibroblast viability compared with either the control or the other composite scaffolds.

### 3.6 Biodegradability

The biodegradability results in PBS at 37°C, calculated based on dry and wet weights using Equation 3‐1, are presented in Table 3. The findings indicate that the mass loss of the PCL nanofiber scaffold increased progressively and uniformly over time.

**Table 3:** Percentage of biodegradability for nanofiber samples.

Day	PCL (*n* = 3) (%)	PCL–collagen 90:10–MTA–TAT (%)
1	5.4 ± 0.03	11 ± 1
7	15.06 ± 0.44	30 ± 2
14	30.2 ± 0.18	50 ± 3

In contrast, the nanofibers composed of PCL–collagen–MTA–TAT exhibited greater degradation on day 7. This pattern may be attributed to the rapid release of collagen and MTA from the nanofibrous structure, as well as enhanced water penetration into the scaffold facilitated by TAT.

Numerous studies in hard tissue engineering have reported that a scaffold mass loss of approximately 10%–30% during the first week is considered optimal for supporting cell proliferation and new ECM formation. The PCL–collagen–MTA–TAT nanofiber scaffold demonstrated a biphasic degradation profile: an initial rapid degradation phase that may facilitate matrix replacement, followed by a slower, more stable phase providing sustained mechanical support. Therefore, this degradation behavior appears favorable for hard tissue engineering applications.

(4‐1) $$\text{Percentage of degradation}\;=\frac{\text{Ww}-\text{Wr}}{\text{Ww}}\times 100.$$

### 3.7 Water Contact Angle

In the analysis of the water droplet contact angle on surfaces based on Young's equation, the contact angle (θ) is defined as the angle formed between the horizontal line and the tangent to the liquid surface at the point of contact with the solid surface. Based on the magnitude of this angle, surfaces are generally classified as follows:

0° < θ < 90°: Hydrophilic

θ = 90°: Neutral wettability

90° < θ < 150°: Hydrophobic

θ > 150°: Superhydrophobic.

**Figure 7:**
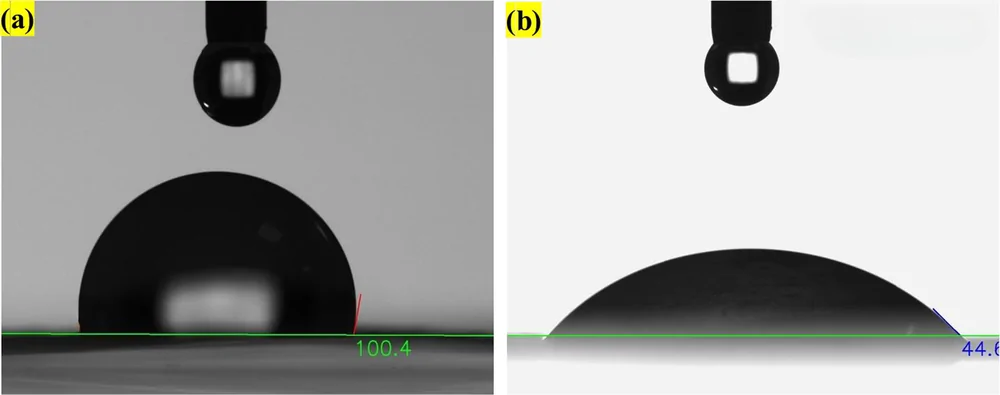
Water contact angle of the PCL–collagen–MTA–TAT nanofiber. (a) Before plasma. (b) After.

To evaluate changes in surface hydrophilicity before and after plasma surface modification, the water contact angle on the nanofiber samples was measured. As shown in Figure 7, the contact angle of the nanofibers before plasma treatment was 100.4°, indicating a hydrophobic surface. After plasma surface modification, the contact angle decreased to approximately 45°, as shown in Figure 7. This reduction indicates a significant increase in the hydrophilicity and surface energy of the sample.

## 4 Discussion

The present study describes the fabrication and initial characterization of an electrospun composite scaffold composed of PCL, collagen, MTA, and TAT peptide. The primary objective was to evaluate its physicochemical properties and short‐term cytocompatibility as a preliminary step toward potential applications in dental tissue engineering. Accordingly, the findings should be interpreted within the scope of an early‐stage, exploratory investigation rather than as definitive evidence of regenerative efficacy.

### 4.1 Electrospun PCL‐Based Scaffolds and ECM Mimicry

Electrospinning enables the production of nanofibrous matrices that reproduce certain architectural aspects of the ECM, particularly fiber scale and porosity. In the present study, SEM analysis demonstrated continuous, bead‐free, randomly oriented nanofibers forming an interconnected porous network. Such morphology is consistent with successful electrospinning and comparable to previously reported PCL‐based nanofibrous scaffolds (Azari et al. 2021; Robles 2024).

In the context of dental and periodontal applications, electrospun nanofibers have been proposed as barrier membranes, drug‐delivery platforms, and regenerative scaffolds. Recent literature describes electrospun membranes with osteogenic, antibacterial, and anti‐inflammatory functionalities for periodontal regeneration, emphasizing the need for bioactive, yet mechanically robust, nanofibrous constructs (Santos et al. 2023; Kalluri et al. 2024). Likewise, electrospun nanofibers have been recognized as potential materials for a range of dental applications, including pulp–dentin complex regeneration, periodontal regeneration, and guided tissue regeneration membranes (Santos et al. 2023; Kalluri et al. 2024). While ECM‐like morphology is often associated with favorable cellular responses, it is important to emphasize that structural similarity alone does not ensure biological functionality. Quantitative evaluation of cell adhesion, infiltration, and matrix deposition was not performed in this study. Therefore, the observed architecture should be considered a supportive structural framework whose biological performance requires further validation.

### 4.2 Role of Collagen and Composite PCL–Collagen Scaffolds

Blending collagen with PCL is a well‐established strategy to enhance hydrophilicity and introduce bioactive motifs that interact with integrin receptors. Previous investigations have reported improved cell attachment and proliferation on PCL–collagen composites compared with pure PCL (Yuwono et al. 2023; Ren et al. 2025). In agreement with these findings, incorporation of collagen in the present study did not adversely affect fibroblast viability (Yuwono et al. 2023).

More recently, Ren et al. used recombinant human‐like collagen blended with PCL to prepare nanofibrous membranes for periodontal tissue repair and reported improved cell responses and regeneration potential (Ren et al. 2025). These findings support our approach of combining PCL and collagen to generate a biologically instructive matrix. The high fibroblast viability observed on our PCL–collagen blends confirms that the addition of collagen does not compromise cytocompatibility and may facilitate subsequent cell adhesion and matrix deposition, which are essential for endodontic and periapical regeneration.

However, the current experimental design did not include direct assays of cell adhesion strength, spreading area, proliferation kinetics, or ECM production. Consequently, although collagen likely contributes biologically relevant motifs, its functional impact within the present scaffold system remains to be quantified through dedicated biological analyses.

### 4.3 MTA as a Bioactive Component in the Scaffold

MTA is well established as a bioactive endodontic material with the capacity to stimulate hard tissue formation and support cell viability. Several in vitro and in vivo studies have shown that MTA exhibits low cytotoxicity and promotes mineralization, osteogenic differentiation, and expression of dentin‐ and bone‐related markers (Tu et al. 2019; Serin Kalay 2019; Lopes et al. 2019; Yamada et al. 2021; Maru et al. 2021; Babaki et al. 2020). Recent systematic reviews concluded that MTA can enhance the osteo/odontogenic potential of mesenchymal stem cells and is a potential biomaterial for stem cell–based endodontic therapies (Maru et al. 2021; Babaki et al. 2020).

Our incorporation of MTA into a PCL–collagen nanofibrous scaffold is consistent with the trend toward combining bioactive calcium silicate cements with polymeric matrices to enhance regenerative potential. Available evidence indicates that MTA‐based materials show favorable interaction with fibroblasts and gingival cells and can support tissue healing without eliciting significant toxicity (Tu et al. 2019; Serin Kalay 2019; Lopes et al. 2019; Yamada et al. 2021; Maru et al. 2021; Babaki et al. 2020). The finding that fibroblast viability remained above 90% on PCL–collagen–MTA scaffolds at both time points suggests that the incorporation of MTA did not introduce significant cytotoxicity under the experimental conditions. This is in agreement with studies showing that MTA eluates and MTA‐based materials are generally well tolerated by fibroblasts and stem cells, while simultaneously stimulating mineralized tissue formation (Tu et al. 2019; Serin Kalay 2019; Lopes et al. 2019; Yamada et al. 2021; Maru et al. 2021; Babaki et al. 2020).

Although our study did not directly assess osteogenic differentiation or mineral deposition, previous reports on MTA‐containing systems indicate that sustained release of calcium and hydroxyl ions can promote apatite formation and upregulate osteogenic markers (Maru et al. 2021; Babaki et al. 2020). Nevertheless, the study did not evaluate calcium ion release, pH changes, apatite formation, or osteogenic/odontogenic differentiation markers. Therefore, although literature supports the bioactivity of MTA, the regenerative functionality of MTA within the current nanofibrous matrix cannot be inferred from the present data. Future work should include ion release profiling, mineralization assays, alkaline phosphatase activity, and gene expression analyses using lineage‐relevant stem cell populations.

### 4.4 TAT Peptide Functionalization and Surface Charge Modulation

Functionalization of biomaterials with bioactive peptides has gained considerable attention as a means to modulate cell adhesion, signaling, and delivery of therapeutic agents. RGD‐containing peptides, for instance, have been used to improve endothelial and osteoblast adhesion to PCL‐based scaffolds (Kumar et al. 2023). CPPs such as TAT extend this concept by enabling efficient transport of biomolecules across cell membranes and by altering the electrostatic interactions at the cell–material interface.

Recent work has shown that scaffolds or nanoparticles functionalized with CPPs can enhance cellular uptake, gene delivery, and multicellular interactions in tissue engineering settings (Raftery et al. 2022). Raftery et al. developed CPP‐loaded scaffolds for localized gene delivery and demonstrated efficient transfection across multiple cell types without compromising cell viability (Raftery et al. 2022). Our zeta potential data, showing a shift from negative to positive values after TAT incorporation, are consistent with the cationic nature of TAT and suggest successful surface functionalization. A positively charged surface is expected to increase electrostatic attraction with negatively charged cell membranes and serum proteins, potentially enhancing cell adhesion and spreading.

Although the present study did not directly compare cell adhesion or proliferation kinetics between TAT‐functionalized and non‐functionalized scaffolds, the absence of cytotoxicity and the favorable surface charge profile support the feasibility of using TAT to tune bioactive interactions further. Future studies should quantify cell adhesion strength, spreading area, and gene delivery potential on TAT‐modified nanofibers, drawing on the growing literature on peptide‐functionalized scaffolds for controlled cell behavior (Raftery et al. 2022; Kumar et al. 2023).

However, several important limitations must be acknowledged. First, the TAT peptide was incorporated through physical blending rather than covalent immobilization. As a result, its surface availability, long‐term stability, and release kinetics were not determined. Second, no direct biological assays were performed to evaluate whether TAT enhanced cell adhesion, internalization processes, or signaling pathways. Therefore, while the observed surface charge shift suggests successful incorporation at a physicochemical level, the biological contribution of TAT remains speculative and requires further experimental verification.

### 4.5 Cytocompatibility and Comparison With Previous PCL‐Based Scaffolds

Fibroblast viability on all scaffold compositions remained above 90% at 24 and 48 h, indicating that the electrospun PCL–collagen–MTA–TAT scaffold is non‐cytotoxic under the conditions tested. Electrospun PCL and PCL/biopolymer scaffolds have repeatedly shown good cytocompatibility with fibroblasts and other cell types in MTT and related assays (Azari et al. 2021; Robles et al. 2024; Yuwono et al. 2023; Ren et al. 2025). Lim et al. and others reported that PCL and PCL/gelatin or PCL–collagen scaffolds supported fibroblast proliferation over several days, with viability close to that of tissue culture plastic (Azari et al. 2021; Robles et al. 2024; Yuwono et al. 2023). Similarly, recent studies on electrospun PCL–collagen or PCL–gelatin composites have demonstrated high viability and favorable morphology of chondrocytes, osteoblasts, and periodontal ligament cells, reinforcing the biocompatibility of these materials (Yuwono et al. 2023; Ren et al. 2025).

Fibroblasts were selected in this study as a sensitive and clinically relevant screening cell type, as they play a key role in wound healing, ECM deposition, and connective tissue remodeling in both periodontal and periapical tissues. In dental materials research, fibroblasts are frequently used as an initial biological model to assess material cytotoxicity and biocompatibility before more specialized differentiation studies. Moreover, fibroblasts are known to respond sensitively to changes in surface chemistry, ion release, and pH, making them suitable for detecting potential adverse effects associated with calcium silicate–based materials such as MTA. The MTT assay was used as a standardized and widely accepted method to evaluate early metabolic activity and cell viability on dental biomaterials. Although MTT primarily reflects mitochondrial activity rather than direct measures of cell adhesion or proliferation, it remains a recommended first‐line assay in biocompatibility screening of novel dental materials and scaffold systems. The consistently high viability observed across all scaffold groups suggests that neither the incorporation of MTA nor the surface functionalization with TAT peptide induced acute cytotoxic responses during early cell–material interaction.

Our findings are in line with these reports and extend them by showing that the simultaneous inclusion of MTA and TAT peptide in a PCL–collagen matrix does not adversely affect fibroblast survival over 48 h. Together with recent dental‐focused reviews on electrospun polymeric nanofibers (Santos et al. 2023; Kalluri et al. 2024), these results suggest that such composite nanofibrous scaffolds can be safely considered for further development in endodontic and periodontal regenerative strategies.

The biodegradability test showed that the PCL scaffolds exhibited a gradual mass loss of 5.4%, 15.6%, and 30% on days 1, 7, and 14, respectively. In contrast, the complete structure lost approximately 11%, 30%, and 50% of its mass over the same time points (Table 2).

According to previous studies, the degradation rate of a scaffold should be compatible with the rate of cell proliferation. If degradation occurs too rapidly, cell proliferation may be disrupted. Conversely, excessively slow degradation can negatively affect the biological performance of cells.

The biphasic degradation profile observed in this scaffold is consistent with the findings reported by Fu et al., who investigated electrospun PCL/gelatin and PCL–collagen nanofiber scaffolds, as well as with the study by Hadi et al., which developed scaffolds composed of PCL, collagen, and hydroxyapatite nanofibers (Hadi et al. 2023; Fu et al. 2014).

In the water contact angle test, the PCL–collagen–MTA–TAT sample exhibited a contact angle of 100.4° (hydrophobic), which decreased to 45° (hydrophilic) after plasma surface modification (Figure 6). This reduction of more than 55° indicates the stabilization of carboxyl and hydroxyl functional groups on the surface, which can form electrostatic interactions with dissolved ions such as calcium (Karimi et al. 2019).

This reduction is consistent with the findings of Esmaeili Ranjbar et al., who reported a decrease in the contact angle from 104° to 32° following surface modification of PCL nanofibers for tissue engineering applications. Similarly, Stastna et al. observed a reduction from 125° to 45° when investigating the biological effects of hydroxyapatite nanoparticles deposited on PCL nanofibers. Such improvements in surface wettability enhance cell adhesion and facilitate mass transfer (Esmaeili Ranjbar et al. 2025; Stastna et al. 2020).

## 5 Limitations and Future Directions

The present study represents an initial investigation focused primarily on the fabrication and preliminary characterization of a composite electrospun scaffold composed of PCL, collagen, MTA, and TAT peptide. While the results demonstrate favorable physicochemical characteristics and short‐term cytocompatibility, several limitations should be acknowledged.

First, the biological evaluation was limited to short‐term cytocompatibility testing using HDFs at 24 and 48 h. Although fibroblasts are commonly used as an initial screening model in biomaterials research due to their sensitivity to material composition and surface properties, they do not fully represent the specialized cell populations involved in dental or craniofacial tissue regeneration. Future studies should therefore investigate cellular responses using more clinically relevant cell types, such as dental pulp stem cells, stem cells from the apical papilla, periodontal ligament stem cells, or osteoblast‐like cells. In addition, longer culture periods and functional assays—including cell adhesion, proliferation kinetics, osteogenic or odontogenic differentiation, and mineralization—will be necessary to assess the biological performance of the scaffold better.

Second, the current study relied primarily on metabolic activity measurements using the MTT assay to evaluate cytocompatibility. Although this assay is widely used for initial biocompatibility screening, it reflects mitochondrial activity rather than direct measures of cell adhesion, proliferation dynamics, or differentiation potential. Complementary biological assays, including live–dead staining, cytoskeletal organization analysis, alkaline phosphatase activity, and gene expression profiling of osteogenic or odontogenic markers, would provide a more comprehensive understanding of scaffold–cell interactions.

Third, although the incorporation of TAT peptide resulted in a measurable change in surface charge, the peptide was introduced through physical blending during electrospinning rather than through covalent immobilization. Consequently, its long‐term stability, surface availability, and release behavior were not evaluated in this study. Future investigations should characterize peptide release kinetics, surface retention, and biological activity to clarify the functional contribution of TAT peptide to cellular responses.

Fourth, the mechanical characterization was limited to uniaxial tensile testing of the final scaffold formulation under dry laboratory conditions. Mechanical testing of additional scaffold compositions, evaluation under physiologically relevant hydrated environments, and comparison with the mechanical properties of native tissues would be necessary to determine the suitability of the scaffold for specific clinical applications.

Finally, several material properties that are important for dental and craniofacial scaffolds were not evaluated in the present work. These include scaffold degradation behavior, ion release from the MTA component, wettability, antibacterial properties, and long‐term structural stability. Assessing these parameters would provide valuable insights into the scaffold's behavior in biological environments and its potential translational relevance.

Despite these limitations, the present study provides a foundational platform for the development of multi‐component electrospun scaffolds integrating polymeric, biological, mineral, and peptide elements. Future studies addressing the aforementioned aspects, particularly through comprehensive biological assays and relevant in vivo models, will be essential to determine the potential applicability of this scaffold in regenerative dental and craniofacial therapies.

Accordingly, the biological evaluation in this study was intentionally limited to an early cytocompatibility assessment, aimed at establishing material safety before more complex functional and differentiation analyses.

## 6 Conclusions

In this study, a composite electrospun nanofibrous scaffold composed of PCL, collagen, MTA, and TAT peptide was successfully fabricated and initially characterized. The scaffold exhibited a porous nanofibrous architecture, s incorporation of its components, and a shift in surface charge following TAT addition, suggesting modification of surface physicochemical properties. Mechanical testing indicated that the scaffold maintained structural integrity under the tested conditions.

Short‐term cytocompatibility assays using HDFs demonstrated high cell viability, indicating that incorporation of collagen, MTA, and TAT peptide did not induce detectable cytotoxic effects.

Overall, the findings provide preliminary evidence that this composite electrospun scaffold can serve as a potential platform for further biomaterials development. However, comprehensive biological studies, including adhesion, differentiation, mineralization, and long‐term performance using relevant dental stem cell models, are necessary to determine its suitability for dental and craniofacial regenerative applications.

## Author Contributions

All authors contributed to the study conception and design. Material preparation, data collection, and analysis were performed by authors. The first draft of the manuscript was written by Ezatolah Kazeminezhad and all authors commented on previous versions of the manuscript. All authors read and approved the final manuscript.

## Funding

The authors have nothing to report.

## Conflicts of Interest

The authors declare no conflicts of interest.

## Data Availability

The data that support the findings of this study are available on request from the corresponding author. The data are not publicly available due to privacy or ethical restrictions. The dataset used and analyzed during the current study is available from the corresponding author on reasonable request.
